# Nomogram for predicting overall survival in colorectal cancer with distant metastasis

**DOI:** 10.1186/s12876-021-01692-x

**Published:** 2021-03-04

**Authors:** Zheng Liu, Yao Xu, Guijun Xu, Vladimir P. Baklaushev, Vladimir P. Chekhonin, Karl Peltzer, Wenjuan Ma, Xin Wang, Guowen Wang, Chao Zhang

**Affiliations:** 1 Department of Bone and Soft Tissue Tumors, Tianjin Medical University Cancer Institute and Hospital, National Clinical Research Center for Cancer, Key Laboratory of Cancer Prevention and Therapy, Tianjin’s Clinical Research Center for Cancer, Tianjin, 300060 China; 2 Department of Orthopedics, Heilongjiang Provincial Hospital, Harbin, Heilongjiang Province China; 3 Department of Orthopaedics, Tianjin Hospital, Tianjin, China; 4 Federal Research and Clinical Center of Specialized Medical Care and Medical Technologies, Federal Biomedical Agency of the Russian Federation, Moscow, Russian Federation; 5 Department of Basic and Applied Neurobiology, Federal Medical Research Center for Psychiatry and Narcology, Moscow, Russian Federation; 6 Department of Research and Innovation, University of Limpopo, Turfloop, South Africa; 7 Department of Breast Imaging, Tianjin Medical University Cancer Institute and Hospital, National Clinical Research Center for Cancer, Key Laboratory of Cancer Prevention and Therapy, Tianjin’s Clinical Research Center for Cancer, Tianjin, China; 8 Department of Epidemiology and Biostatistics, First Affiliated Hospital, Army Medical University, Chongqing, China; 9 Sino-Russian Joint Research Center for Bone Metastasis in Malignant Tumor, Tianjin, China

**Keywords:** Colorectal neoplasms, Stage IV, Overall survival, Nomograms, SEER program

## Abstract

**Background:**

Colorectal cancer (CRC) is a major cancer burden, and prognosis is determined by many demographic and clinicopathologic factors. The present study aimed to construct a prognostic nomogram for colorectal cancer patients with distant metastasis.

**Methods:**

Colorectal cancer patients with distant metastasis diagnosed between 2010 and 2016 were selected from the Surveillance, Epidemiology, and End Results database. Cox proportional hazards regression was used to identify independent prognostic factors. A nomogram was constructed to predict survival, and validation was performed.

**Results:**

A total of 7099 stage IV colorectal cancer patients were enrolled in the construction cohort. The median overall survival was 20.0 (95% CI 19.3–20.7) months. Age at diagnosis, marital status, race, primary tumour site, tumour grade, CEA level, T stage, N stage, presence of bone, brain, liver and lung metastasis, surgery for primary site and performance of chemotherapy were independent prognostic factors. The nomogram was constructed and the calibration curve showed satisfactory agreement. The C-index was 0.742 (95% CI 0.726–0.758). In the validation cohort (7098 patients), the nomogram showed satisfactory discrimination and calibration with a C-index of 0.746 (95% CI 0.730–0.762).

**Conclusion:**

A series of factors associated with the survival of CRC patients with distant metastasis were found. Based on the identified factors, a nomogram was generated to predict the survival of stage IV colorectal cancer patients. The predictive model showed satisfactory discrimination and calibration, which can provide a reference for survival estimation and individualized treatment decisions.

## Background

Colorectal cancer (CRC) is the third most frequent cancer and one of the main causes of cancer-related death [1]. In 2016, there were 1,700,000 new CRC cases and 830,000 deaths attributed to CRC worldwide [2]. Although treatment strategies, such as immunotherapy, chemotherapy and targeted agents, have been developing rapidly in recent decades, the prognosis of CRC is still unsatisfactory [3–5]. It was reported that the 5-year overall mortality rate was 65.0–70.0% for stage III CRC patients [6, 7]. Compared to patients without metastasis, the survival outcome of metastatic CRC patients was worse, with a 5-year survival rate of only 14.0% [8]. A previous study analysed 374 stage IV CRC patients and suggested that synchronous metastatic CRC patients had worse 3-year survival (33.0%) than metachronous CRC patients (54.0%, *P* = 0.0038) [9]. The incidence of synchronous distant metastasis was reported to be increasing in the latest study, with rates of 15%-20% in CRC patients [10, 11]. Survival of patients with distant metastasis significantly affects the organization of individualized treatment. Thus, studies focusing on survival estimation of initial stage IV CRC are urgently needed.

Various prognostic factors for stage IV CRC patients have been investigated in previous studies. Several demographic and clinicopathologic variables were proven to be independent prognostic factors: age at diagnosis [11], tumour size [12], lymph metastasis [12], resectability [12], and chemotherapy treatment [10]. Carcinoembryonic antigen (CEA) levels and primary tumour sites were reported to be associated with the survival of CRC [9, 13]. In a multicentre register study comprising 9624 stage IV CRC patients, a prognostic scoring system was established based on eight independent prognostic factors. The total score was ranged from 0 to 9, and higher scores indicated poorer survival [9, 13].

Nomograms are widely used as graphical prediction models, by which survival predictive points can be calculated based on the predictors [15]. Several prognostic nomograms for stage IV CRC have been constructed in recent years [16–18]. Based on 1133 stage IV CRC patients who received curative resection, nomograms to predict disease-free survival (DFS) and overall survival (OS) were constructed [16]. However, only four predictors (T stage, N stage, postoperative CEA and metastatic organs) were included in the models, which led to potential inaccuracy. In 2019, Hua Ge et al. reported a nomogram to predict OS in CRC patients at M1 stage [17]. Several social characteristics and tumour-related variables were included in the nomogram. Nevertheless, neither serum CEA level nor the performance of adjuvant treatment (radiation and chemotherapy) were included as predictors, which are widely considered independent prognostic factors for CRC patients [13]. Another SEER-based study enrolled 2996 CRC patients with stage IV disease, and a predictive nomogram was constructed [18]. External validation was performed based on a Chinese cohort with high discrimination (C-index of OS: 0.657; 95% CI 0.544–0.770), which indicated good transportability of the nomogram [18]. However, the nomogram was used to predict the survival of patients who underwent both primary and metastatic resection. A nomogram including as many necessary predictors as possible is urgently needed to accurately estimate the current survival of stage IV CRC.

Extracting data from the Surveillance Epidemiology and End Results (SEER) database, the present study aimed to identify prognostic factors for CRC patients with distant metastasis. The factors were then used to construct a prognostic prediction nomogram. The predictive model can help oncologists accurately estimate prognosis and guide the individualized treatment.

## Methods

### Data source and cohort selection

Data were extracted from the Surveillance Epidemiology and End Results (SEER) database of the National Cancer Institute (https://seer.cancer.gov/), which covers approximately 30% of the US population. The SEER program provides information on cancer statistics to reduce cancer burden and all authors are permitted to access the original data without informed consent. The present study complied with the 1964 Declaration of Helsinki and its later amendments or comparable ethical standards.

**Fig. 1:**
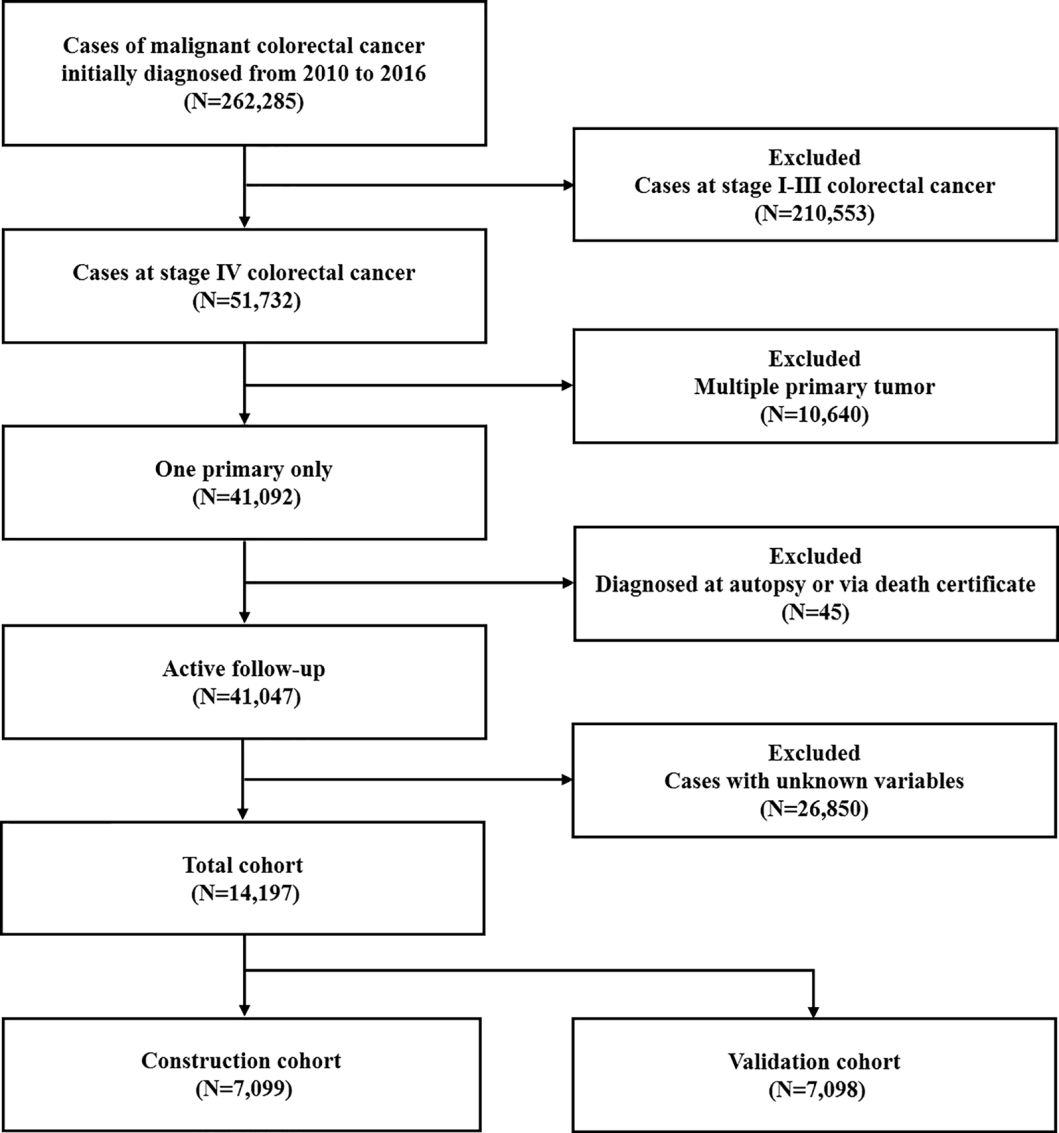
The detailed flow-chart for patient selection

The database, which was *Incidence—SEER 18 Regs Custom Data (with additional treatment fields), Nov 2018 Sub (1975–2016 varying)*, was released in April 2019 and was selected as the data source for the present study. Patients with malignant colorectal cancer were extracted from the database according to the *Site recode ICD-O-3/WHO 2008* of ‘Colon and Rectum’ and the *Behavior recode for analysis* of ‘Malignant’. The year of diagnosis was restricted between 2010 and 2016 since data involving metastatic sites were not available until 2010. The exclusion criteria were as follows: (1) colorectal cancer cases from stage I-III; (2) two or more primary tumours; (3) diagnosed at autopsy or via death certificate; and (4) cases with unknown information about demographic and clinicopathologic variables. A detailed flow-chart for patient selection is shown in Fig. 1.

### Demographic and clinicopathologic variables

The following demographic and clinicopathologic variables were included in the present study: age at diagnosis (< 65 and ≥ 65 years), gender (male and female), marital status (married and unmarried), race (white, black and others), insurance status (insured and uninsured), primary site (left colon, right colon and other sites), tumour grade (I to IV: well, moderately, poorly and undifferentiated, respectively), carcinoembryonic antigen (CEA) level (normal and elevated), T stage (T1, T2, T3 and T4), N stage (N0, N1 and N2), the presence of bone, brain, liver, lung metastasis (no, yes), surgery for primary site (no, yes), radiation treatment (no/unknown, yes) and the performance of chemotherapy (no/unknown, yes). According to the *Primary Site – labeled*, the primary site was divided into ‘Left colon’ (C18.5-Splenic flexure of colon, C18.6-Descending colon, C18.7-Sigmoid colon, C19.9-Rectosigmoid junction and C20.9-Rectum, NOS), ‘Right colon’ (C18.0-Cecum, C18.1-Appendix, C18.2-Ascending colon, C18.3-Hepatic flexure of colon and C18.4-Transverse colon) and ‘Other sites’ (C18.8-Overlapping lesion of colon and C18.9-Colon, NOS) categories.

### Statistical analysis

In the present study, the total cohort was randomly subdivided into construction and validation cohorts (ratio 1:1). A construction cohort was used to identify prognostic factors for stage IV colorectal cancer patients, and a nomogram was constructed, while the validation cohort was used to validate the performance of the model. Quantitative data are described as the mean ± standard deviation (SD), while categorical variables are presented as numbers and percentages (N, %). The primary outcome was overall survival (OS), which was defined as the time from diagnosis of colorectal cancer to all causes of death. Cox proportional hazards regression was performed to identify prognostic factors. Variables with significant differences in the univariate analysis were further analysed with a multivariate analysis to determine the independent prognostic factors. Based on the prognostic factors, the nomogram was formulated using the survival package in R. Each predictor included in the nomogram was represented on one row, and a corresponding number of points was assigned to different magnitudes of the predictor. The cumulative point axis was represented at the end of the nomogram, and higher total points indicated a worse survival outcome. The discriminative ability of the model was evaluated with Harrell's concordance index (C-index) and receiver operating characteristic (ROC) curve analysis. A larger C-index value and a greater area under the curve (AUC) in the ROC curve indicated better discrimination ability. Calibration curves (1000 bootstrap resamples) were generated to evaluate the calibration ability of the nomogram.

The case listing session of the SEER*Stat 8.3.6 program was used to generate data and IBM SPSS Statistics (version 26.0, Armonk, NY, USA) was used for statistical analyses. The construction of the prognostic nomogram and subsequent validation were performed with R version 4.0.0 (R Foundation for Statistical Computing, Vienna, Austria; www.r-project.org). All statistical tests were two-sided, and *P* < 0.05 was considered significant.

## Results

### Demographic and clinicopathologic characteristics

According to the inclusion and exclusion criteria, a total of 7099 patients with stage IV colorectal cancer were included in the construction cohort. The mean age was 61.5 ± 13.7 years, with a slight predominance for male (N = 3782, 53.3%) and married (N = 3880, 54.7%) patients. The majority of the construction population was white (N = 5358, 75.8%) and insured (N = 6755, 95.2%). Tumour grade I, grade II, grade III and grade IV accounted for 5.4%, 64.5%, 25.1% and 5.0%, respectively. More than half of tumours (N = 3948, 55.6%) occurred in the left colon, while 42.3% were located in the right colon. The number of cases with elevated CEA was 5604, accounting for 78.9% in the construction cohort. T3 stage (N = 3362, 47.4%) was the most common tumour stage, followed by T4 stage (N = 2885, 40.6%), T1 stage (N = 653, 9.2%) and T2 stage (N = 199, 2.8%). The percentage of patients with lymph node metastasis was 74.2%. There were 284, 78, 5000 and 1458 patients diagnosed with bone, brain, liver and lung metastasis, respectively. Regarding the treatment strategy, nearly 80% of patients underwent surgery for colorectal tumour sites. Radiation and chemotherapy were administered to 939 and 5299 patients, respectively. Detailed information about the demographic and clinicopathologic characteristics of the validation cohort is shown in Table 1.

**Table 1:** Baseline demographic and clinicopathologic characteristics in the construction and validation cohort

Subject characteristics	Construction cohort (N = 7099)		Validation cohort (N = 7098)	
	Alive (N, %)	Dead (N, %)	Alive (N, %)	Dead (N, %)
Age (years, mean = 61.5 ± 13.7; median = 61)				
< 65	1679 (67.6)	2523 (54.7)	1765 (68.2)	2433 (53.9)
≥ 65	804 (32.4)	2093 (45.3)	823 (31.8)	2077 (46.1)
Gender				
Male	1295 (52.2)	2487 (53.9)	1329 (51.4)	2431 (53.9)
Female	1188 (47.8)	2129 (46.1)	1259 (48.6)	2079 (46.1)
Marital status				
Married	1494 (60.2)	2386 (51.7)	1568 (60.6)	2372 (52.6)
Unmarried	989 (39.8)	2230 (48.3)	1020 (39.4)	2138 (47.4)
Race				
White	1885 (75.9)	3473 (75.2)	1983 (76.6)	3436 (76.2)
Black	323 (13.0)	740 (16.0)	331 (12.8)	665 (14.7)
Others	275 (11.1)	403 (8.7)	274 (10.6)	409 (9.1)
Insurance				
Insured	2398 (96.6)	4357 (94.4)	2457 (94.9)	4253 (94.3)
Uninsured	85 (3.4)	259 (5.6)	131 (5.1)	257 (5.7)
Primary site				
Left colon	1525 (61.4)	2423 (52.5)	1610 (62.2)	2335 (51.8)
Right colon	915 (36.9)	2089 (45.3)	943 (36.4)	2056 (45.6)
Other sites	43 (1.7)	104 (2.3)	35 (1.4)	119 (2.6)
Grade				
Grade I	160 (6.4)	222 (4.8)	181 (7.0)	198 (4.4)
Grade II	1792 (72.2)	2790 (60.4)	1852 (71.6)	2787 (61.8)
Grade III	445 (17.9)	1338 (29.0)	452 (17.5)	1248 (27.7)
Grade IV	86 (3.5)	266 (5.8)	103 (4.0)	277 (6.1)
CEA				
Normal	661 (26.6)	834 (18.1)	748 (28.9)	853 (18.9)
Elevated	1822 (73.4)	3782 (81.9)	1840 (71.1)	3657 (81.1)
T stage				
T1	140 (5.6)	513 (11.1)	154 (6.0)	493 (10.9)
T2	103 (4.1)	96 (2.1)	88 (3.4)	97 (2.2)
T3	1328 (53.5)	2034 (44.1)	1390 (53.7)	1992 (44.2)
T4	912 (36.7)	1973 (42.7)	956 (36.9)	1928 (42.7)
N stage				
N0	670 (27)	1159 (25.1)	703 (27.2)	1058 (23.5)
N1	1008 (40.6)	1687 (36.5)	1066 (41.2)	1643 (36.4)
N2	805 (32.4)	1770 (38.3)	819 (31.6)	1809 (40.1)
Bone metastasis				
No	2434 (98.0)	4381 (94.9)	2541 (98.2)	4282 (94.9)
Yes	49 (2.0)	235 (5.1)	47 (1.8)	228 (5.1)
Brain metastasis				
No	2468 (99.4)	4553 (98.6)	2574 (99.5)	4457 (98.8)
Yes	15 (0.6)	63 (1.4)	14 (0.5)	53 (1.2)
Liver metastasis				
No	809 (32.6)	1290 (27.9)	887 (34.3)	1188 (26.3)
Yes	1674 (67.4)	3326 (72.1)	1701 (65.7)	3322 (73.7)
Lung metastasis				
No	2061 (83.0)	3580 (77.6)	2166 (83.7)	3526 (78.2)
Yes	422 (17.0)	1036 (22.4)	422 (16.3)	984 (21.8)
Surgery for primary site				
No	379 (15.3)	1235 (26.8)	418 (16.2)	1152 (25.5)
Yes	2104 (84.7)	3381 (73.2)	2170 (83.8)	3358 (74.5)
Radiation				
No/unknown	2102 (84.7)	4058 (87.9)	2189 (84.6)	3986 (88.4)
Yes	381 (15.3)	558 (12.1)	399 (15.4)	524 (11.6)
Chemotherapy				
No/unknown	321 (12.9)	1479 (32)	305 (11.8)	1458 (32.3)
Yes	2162 (87.1)	3137 (68)	2283 (88.2)	3052 (67.7)

### Survival and prognostic factors of stage IV colorectal cancer

A total of 4616 patients decreased in the construction cohort and the median overall survival (OS) was 20.0 (95% CI 19.3–20.7) months. The 1-, 3- and 5-year OS rates were 64.8%, 28.7% and 15.4%, respectively. In the univariate Cox regression analysis, the following variables were associated with survival: age at diagnosis, marital status, race, insurance status, primary site, tumour grade, CEA level, T stage, N stage, the presence of bone, brain, liver, lung metastasis, surgery for primary site, radiation treatment and chemotherapy treatment. The multivariate analysis identified that age older than 65 years, unmarried status, black race, primary site on the right colon or other sites, higher tumour grade, elevated CEA level, lower T stage, higher N stage, the presence of bone, brain, liver and lung metastasis, no surgery for the primary site and no/unknown performance of chemotherapy were independent prognostic factors for worse survival. More details about the Cox proportional hazard regression are listed in Table 2.

**Table 2:** Cox proportional hazard regression model for analyzing the prognostic factors for colorectal cancer patients at IV stage

Subject characteristics	Univariate		Multivariate	
	HR (95% CI)	*P* value	HR (95% CI)	*P* value
Age				
< 65	1 (reference)	1.00	1 (reference)	1.00
≥ 65	1.60 (1.51–1.70)	< 0.001	1.37 (1.29–1.46)	< 0.001
Gender				
Male	1 (reference)	1.00	1 (reference)	
Female	0.98 (0.92–1.04)	0.478	-	-
Marital status				
Married	1 (reference)	1.00	1 (reference)	1.00
Unmarried	1.34 (1.26–1.42)	< 0.001	1.20 (1.13–1.27)	< 0.001
Race				
White	1 (reference)	1.00	1 (reference)	1.00
Black	1.08 (0.99–1.17)	0.070	1.12 (1.03–1.22)	0.005
Others	0.89 (0.81–0.99)	0.034	0.86 (0.77–0.95)	0.003
Insurance				
Insured	1 (reference)	1.00	1 (reference)	1.00
Uninsured	1.25 (1.10–1.42)	0.001	1.13 (0.99–1.29)	0.066
Primary site				
Left colon	1 (reference)	1.00	1 (reference)	1.00
Right colon	1.35 (1.27–1.43)	< 0.001	1.26 (1.18–1.35)	< 0.001
Other sites	1.55 (1.27–1.88)	< 0.001	1.35 (1.10–1.64)	0.003
Grade				
Grade I	1 (reference)	1.00	1 (reference)	1.00
Grade II	1.05 (0.92–1.21)	0.463	1.08 (0.94–1.23)	0.303
Grade III	1.73 (1.50–1.99)	< 0.001	1.70 (1.48–1.97)	< 0.001
Grade IV	1.80 (1.50–2.15)	< 0.001	1.93 (1.61–2.32)	< 0.001
CEA				
Normal	1 (reference)	1.00	1 (reference)	1.00
Elevated	1.46 (1.35–1.57)	< 0.001	1.39 (1.29–1.50)	< 0.001
T stage				
T1	1 (reference)	1.00	1 (reference)	1.00
T2	0.41 (0.33–0.51)	< 0.001	0.66 (0.53–0.83)	< 0.001
T3	0.54 (0.49–0.60)	< 0.001	0.84 (0.75–0.95)	0.004
T4	0.77 (0.70–0.85)	< 0.001	1.11 (0.99–1.25)	0.071
N stage				
N0	1 (reference)	1.00	1 (reference)	1.00
N1	0.94 (0.87–1.01)	0.095	1.16 (1.07–1.26)	< 0.001
N2	1.15 (1.07–1.24)	< 0.001	1.55 (1.43–1.69)	< 0.001
Bone metastasis				
No	1 (reference)	1.00	1 (reference)	1.00
Yes	2.02 (1.77–2.30)	< 0.001	1.57 (1.37–1.80)	< 0.001
Brain metastasis				
No	1 (reference)	1.00	1 (reference)	1.00
Yes	1.98 (1.54–2.54)	< 0.001	1.44 (1.11–1.86)	0.005
Liver metastasis				
No	1 (reference)	1.00	1 (reference)	1.00
Yes	1.12 (1.05–1.19)	0.001	1.34 (1.25–1.43)	< 0.001
Lung metastasis				
No	1 (reference)	1.00	1 (reference)	1.00
Yes	1.37 (1.28–1.47)	< 0.001	1.30 (1.21–1.4)	< 0.001
Surgery for primary site				
No	1 (reference)	1.00	1 (reference)	1.00
Yes	0.53 (0.49–0.56)	< 0.001	0.43 (0.39–0.47)	< 0.001
Radiation				
No/unknown	1 (reference)	1.00	1 (reference)	1.00
Yes	0.81 (0.74–0.88)	< 0.001	0.95 (0.86–1.04)	0.255
Chemotherapy				
No/unknown	1 (reference)	1.00	1 (reference)	1.00
Yes	0.34 (0.32–0.36)	< 0.001	0.35 (0.32–0.37)	< 0.001

*HR* hazard ratio, *CI* confidence interval

### Construction and validation of the nomogram

**Fig. 2:**
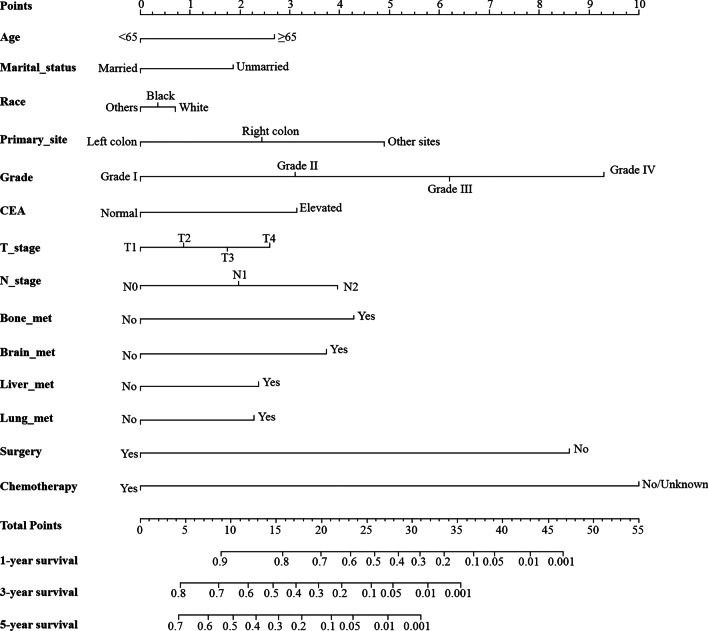
The nomogram for predicting 1-year, 3-year and 5-year survival for colorectal cancer patients with distant metastasis

**Fig. 3:**
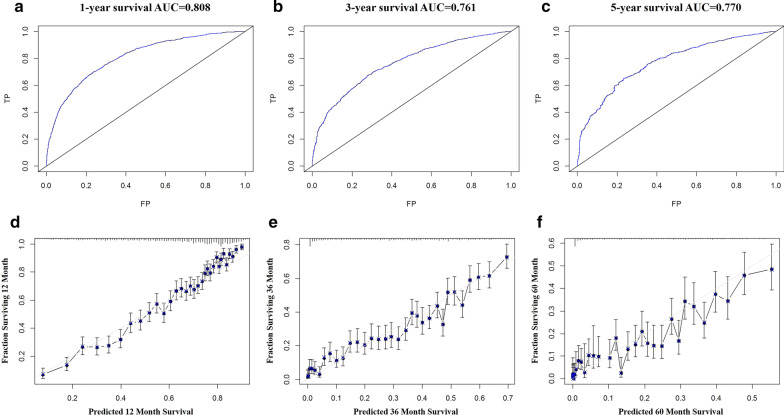
The ROC curve (**a**–**c**) and calibration curve (**d**–**f**) for assessing the discrimination and calibration of the nomogram in construction cohort

As shown in Fig. 2, the nomogram for predicting 1-, 3- and 5-year survival was constructed based on the abovementioned prognostic factors. The C-index for the prediction of OS was 0.742 (95% CI 0.726–0.758), and the AUCs of the nomogram for 1-year, 3-years and 5-years were 80.8%, 76.1% and 77.0%, respectively (Fig. 3a–c). The calibration curve revealed good agreement between the predicted and observed probabilities. All calibration curves were close to the 45-degree line (Fig. 3d–f for 1-year, 3-years and 5-years, respectively).

**Fig. 4:**
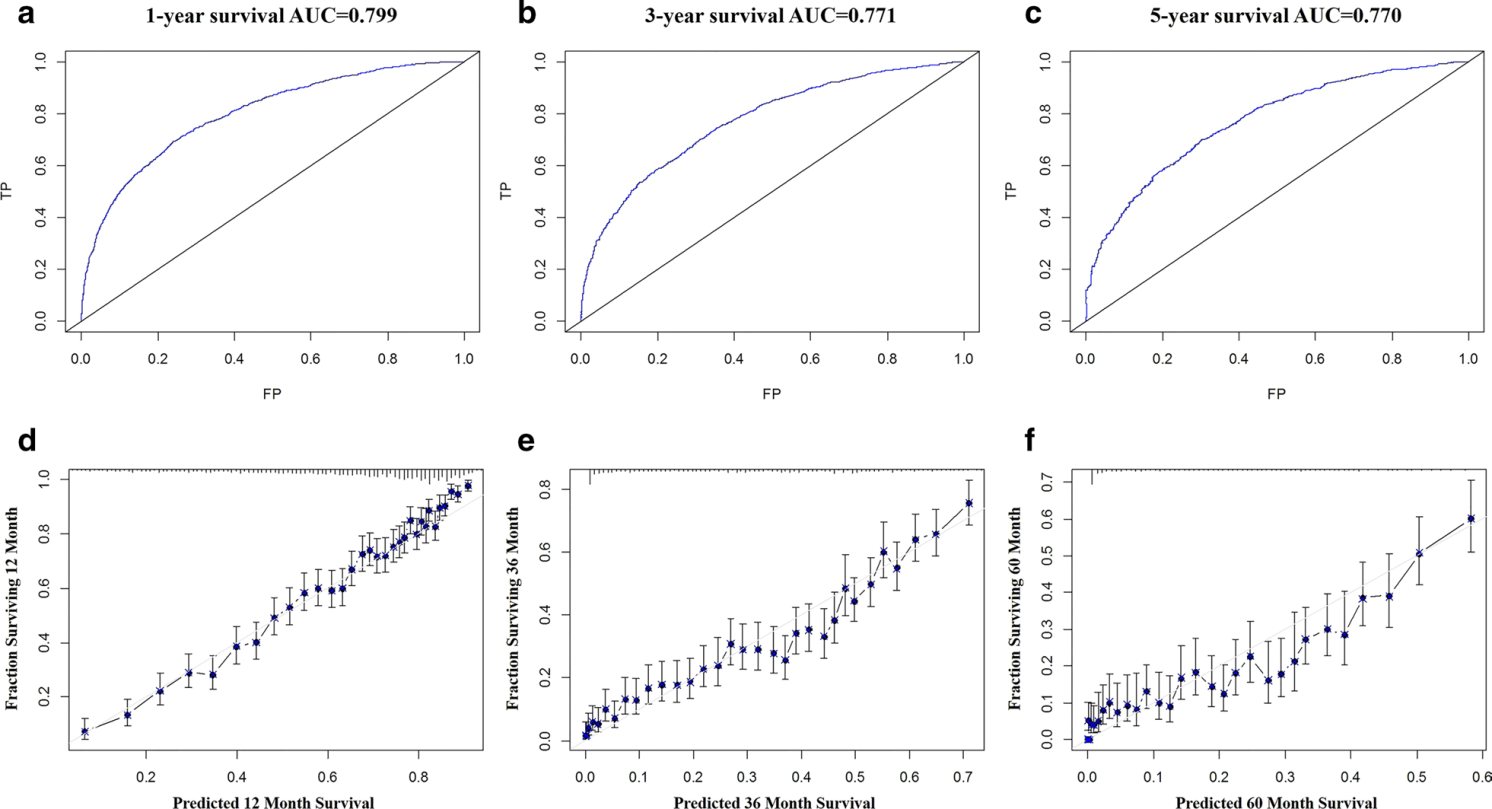
The ROC curve (**a**–**c**) and calibration curve (**d**–**f**) for assessing the discrimination and calibration of the nomogram in validation cohort

In the validation cohort, the nomogram showed satisfactory discrimination strength. The C-index was 0.746 (95% CI 0.730–0.762), and the AUCs for 1-year, 3-year and 5-year survival were 79.9%, 77.1% and 77.0%, respectively (Fig. 4a–c). Excellent calibration ability was achieved with all calibration curves close to the 45-degree line (Fig. 4d–f for 1-year, 3-years and 5-years, respectively).

## Discussion

In the present study, the demographic and clinicopathologic characteristics of stage IV colorectal cancer were described and the survival outcome was estimated. Previous data from four national colorectal cancer registers showed that the 3-year net survival rates of stage IV CRC patients were 20.5%-33.0% and 26.7%-38.5% for colon and rectal cancer, respectively[19]. Another single institution study reported that the 5-year OS was 19.1% for stage IV CRC [12]. Our study observed similar survival rates with the previous studies.

According to the Japanese Society for Cancer of the Colon and Rectum guidelines, resectability should be first considered when making clinical decisions [20]. Systematic chemotherapy and radiotherapy are recommended in unresectable CRC cases, while palliative care is encouraged for patients with end-stage disease [20]. Despite different treatments for patients at different stages, the guidelines do not clearly state the survival estimation for each patient. Undoubtedly, accurate survival estimation is a prerequisite for selecting the aforementioned clinical strategy. A total of fourteen independent prognostic factors were identified in the current study, and a predictive nomogram was constructed based on these predictors. The nomogram presented good discrimination and calibration in the validation cohort.

In the present study, chemotherapy was one of the predictors in the constructed nomogram, which was not previously included [16–18]. According to the Japanese Society for Cancer of the Colon and Rectum guidelines, postoperative adjuvant chemotherapy is recommended in patients with R0 resection [20]. In a retrospective study with 37.0 months of median follow-up, OS rates were 62.1% and 40.4% for CRC patients with and without adjuvant chemotherapy, respectively [13]. Another multi-institutional analysis reported that fewer recurrences were found in patients who received preoperative chemotherapy [10]. In addition, surgery for the primary site was another independent prognostic factor for stage IV CRC. Consistent with our study, no surgery was associated with a 2.807-fold increased risk of death in a previous study [17]. Compared to conventional open surgery, laparoscopic surgery showed advantages of reduced blood loss and shorter hospital stay [21]. No survival difference was found between the two surgical methods [22]. However, information on surgical methods and associated complications was not available in the SEER database.

Tumour grade was the most sensitive predictor in the current study, which was inconsistent with a previous SEER study [18]. This discrepancy may be attributed to the different study populations. In the previous study, only stage IV CRC patients who underwent primary and metastatic resection were included. However, all stage IV CRC patients were selected for the present study. The latest study comprising 126 CRC patients with distant metastasis concluded that grade classification was an independent prognostic factor [12]. Compared to patients with differentiated histology, the hazard ratio for patients with undifferentiated histology was 3.226 (95% CI 1.558–6.711). The 5-year OS rates for patients with differentiated and undifferentiated histology were 21.1% and 11.1%, respectively [12]. There is growing evidence that the primary tumour site is associated with survival of CRC patients. The outcome of patients with tumours in the left colon was better compared to patients with tumours in the right colon [9, 23]. A retrospective SEER dataset reported that the right colon was more likely to present higher T stage and worse histology [23]. Another study concluded that there was increased expression of BRAF mutations in patients with right colon cancer, which was associated with worse survival [24]. The same trend was observed in the present study. The predictive model constructed by Hua Ge et al. indicated that survival of patients with tumour located in the rectum was better compared to patients with tumour located in other sites [17]. In the present study, the “Rectum” site was incorporated into “Left colon”. We did not specifically analyse the survival outcome for patients with a tumour location in the rectum. Compared to the survival of patients with tumour located in different sites, our study suggested that patients with tumour located on overlapping lesions or with undetermined sites exhibited the worst survival.

In the present study, T stage, N stage and the presence of metastasis were proven to be prognostic factors. Thus, these factors were selected into the nomogram. As previously reported, these factors are widely accepted in various cancer prediction models [25, 26]. Consistent with other predictive models, higher T stage, higher N stage and increased sites of metastases indicated worse survival. Furthermore, several previous studies reported that elevated CEA levels indicated poor survival in CRC patients [12, 16]. The aforementioned variables were incorporated into the predictive nomogram of CRC patients [14, 27].

There are some limitations in the current study. First, chemotherapy and radiation information in SEER is incomplete. Further study looking into chemotherapy and radiation on prognosis in stage IV colorectal cancer should be performed. Second, only internal validation was performed. The transportability of nomogram in additional patient populations should be validated in the future. Furthermore, detailed information about treatment, including surgical methods, chemotherapy regimens and targeted agents was not available in the current SEER database, and was reported to be associated with survival [22, 28]. Third, the present real-world study may overestimate the effect of the treatment (especially surgery) on the prognosis. To avoid the selection bias, a further randomized controlled trial on surgery should be performed to quantify such effect. Last but not least, cases with missing data were excluded. This may lead to the reduction of sample’s representativeness. There could be substantial missing data bias due to the amount of missing data. The results might be different if there can be more complete data. Our study developed the auxiliary modelling for prognostic prediction in stage IV colorectal cancer. Such auxiliary tool should be carefully used based on the comprehensive situation of the patients.

## Conclusions

A prognostic nomogram for patients with stage IV colorectal cancer was constructed. The predictive model presented satisfactory discrimination and calibration, which can be used for survival estimation and individualized treatment decision-making in CRC patients with distant metastasis.

## Data Availability

The datasets generated and analysed during the current study are available in the Surveillance, Epidemiology, and End Results database, [https://seer.cancer.gov/]. The database, which named as *Incidence—SEER 18 Regs Custom Data (with additional treatment fields), Nov 2018 Sub (1975-2016 varying),* was released in April 2019 and selected as the data source for the present study. The exclusion and inclusion criteria were stated in the section of Methods. The datasets are available from the corresponding author on reasonable request.

## Abbreviations

CRC: Colorectal cancer
CEA: Carcinoembryonic antigen
DFS: Disease-free survival
OS: Overall survival
SEER: Surveillance Epidemiology and End Results
SD: Standard deviation
ROC: Receiver operating characteristic
AUC: Area under the curve
HR: Ratio
CI: Confidence interval
